# Editorial: Alternative Regenerative Medicine for Diabetes: Beyond the Stem Cell Approach

**DOI:** 10.3389/fendo.2021.648763

**Published:** 2021-07-09

**Authors:** Andrea Peloso

**Affiliations:** Department of Visceral and Transplantation Surgery, University of Geneva Hospitals, Geneva, Switzerland

**Keywords:** regenerative medicine, pancreas bioengineering, diabetes, type 1 diabetes mellitus, organ bioengineering and regeneration

## Introduction

Despite increasing awareness of the problem, diabetes still presents a challenge not only for patients but also for clinicians and other professionals who have to diagnose, monitor, and treat it knowing its many facets. In the last few decades, diabetes has become rampant in the world with unstoppable growth everywhere but especially in developed countries, coupled and supported by an escalating global epidemic of overweight and obesity - (globesity) which is taking over many parts of the world (1). Insulin daily use is the most widespread treatment for type 1 diabetes mellitus (T1DM) while oral hypoglycemics are the first choice in the treatment of type 2 diabetes (T2D). Although these measures have revolutionized diabetes management, they remain sub-optimal treatments. To date, beta cell replacement through pancreas or islets transplantation is considered the only definitive treatment bringing diabetic patients to independence from exogenous insulin administration and increasing both survival and quality of life. However, this solution has several limitations notably the shortage of organs or the need for chronic immunosuppressive therapies that are not without risk.

In this context, dynamic developments in the field of regenerative medicine open up new horizons and bring new hopes in the treatment of diabetes.

This Research Topic draws together two original articles (Penaforte-Saboia, Montenegro et al.; Penaforte-Saboia, Couri et al.), one review article (Peloso et al.) and one commentary article (Cobianchi et al.) in order to explore potential alternative strategies for T1DM.

As one of the leading groups in autologous non-myeloablative hematopoietic stem-cell transplantation (AHST) in T1DM, Penaforte-Saboia et al. assessed the long-term frequency of microvascular complications, beta cell function, and glycemic control in a group of 24 T1DM patients after AHST. Results were associated with data obtained from 144 aged and gender-matched diabetic patients, treated with conventional therapy (CT). In their analysis, the occurrence of microvascular manifestations in transplanted versus CT patients, was lower while a higher residual beta cell function and better glycemic control compared to the CT group was detected.

The same group investigated, in a cross-sectional study, the correlations between insulin dose-adjusted A1c (IDAA1c) (intended as an easy and fast alternative to evaluate pancreatic β-cell function) and microvascular complications (MC), including diabetic retinopathy, neuropathy, and nephropathy (Penaforte-Saboia et al.). The authors report that, in a representative Brazilian population of T1DM patients, those with IDAA1c ≤ 9 presented a lower frequency of MC, as well as fewer episodes of hypoglycemia, in the month prior to the analysis. To our knowledge this is one of the first articles correlating IDAA1c with diabetic microvascular complications and hypoglycemic events in patients with T1DM, which includes a large number of patients undergoing immunomodulatory therapy with autologous non-myeloablative hematopoietic stem-cell transplantation. In total, 144 diabetic patients received conventional therapy (CT) and 24 patients were treated with autologous nonmyeloablative hematopoietic stem-cell transplantation (AHST). These two groups were further subdivided according to their IDAA1c values (30 patients had IDAA1c ≤9; 138 had IDAA1c >9). Then, the prevalence of MC and hypoglycemia were compared between the groups. Finally in this representative population of T1DM patients, patients with IDAA1c ≤ 9 presented a lower frequency of MC, as well as fewer episodes of hypoglycemia, in the month prior to the analysis.

In our review (Peloso et al.), we provided an overview of the existing knowledge of current experimental strategies in the treatment of diabetes covered by the umbrella of regeneration. In particular, stem cell reprogramming, extracellular matrix (ECM) scaffolds, and insulin-like organoid technology have the potential to overcome the actual limitations related to pancreas transplantation. Through an unceasing deep understanding of beta cell and pancreatic development, stem cell research continues to garner interest as a future cure of T1DM. Induced pluripotent stem cells (iPS) are demonstrated to be a potential unprecedented beta cell source *via* their capacity to differentiate into all major somatic cell lineages (Peloso et al.). In the last decade, several natural- and synthetic-derived polymers have been reported as a scaffold solution for organ bioengineering and regenerative medicine (OB/RM) approaches for the treatment of juvenile (type 1) diabetes mellitus. In particular, ECM-derived scaffolds have been extensively explored, showing their potential to be merged to a beta cell source to address the shortage of transplantable organs. This cell-on-scaffold technology stems from the ability to remove cells from a tissue, leaving the ECM almost intact in terms of tridimensional architecture and biological cues.

Finally the commentary offered by Cobianchi et al. covers one of the most promising approaches for beta-cell replacement: the 3D cell aggregate system. This system has been used by Lebreton et al. to manufacture insulin-producing engineered organoids (2). The commentary highlights the importance of the use of dissociated islands and human amniotic epithelial cells (hAECs); a breakthrough in this field.

In conclusion, the articles in this Research Topic not only provide important insights concerning T1DM (Penaforte-Saboia, Montenegro et al.; Penaforte-Saboia, Couri et al.), but also explore new strategies for beta cell replacement (Peloso et al.; Cobianchi et al.) (2).

## Author Contributions

AP edited and revised the manuscript. The author confirms being the sole contributor of this work and has approved it for publication. 

## Conflict of Interest

The author declares that the research was conducted in the absence of any commercial or financial relationships that could be construed as a potential conflict of interest.

## Abbreviations

T1DM, type 1 diabetes mellitus; T2D, type 2 diabetes; AHST, autologous non-myeloablative hematopoietic stem-cell transplantation; IDAA1c, insulin dose adjusted A1c; MC, microvascular complications; ECM, extracellular matrix; iPS, Induced pluripotent stem cells; OB/RM, organ bioengineering and regenerative medicine.
